# Supplementary figures and images for: Deterioration of postural control due to the increase of similarity between center of pressure and smooth-pursuit eye movements during standing on one leg
Source: PLoS One. 2022 Oct 13;17(10):e0276119. doi: 10.1371/journal.pone.0276119 (PMC9560487; doi:10.1371/journal.pone.0276119)

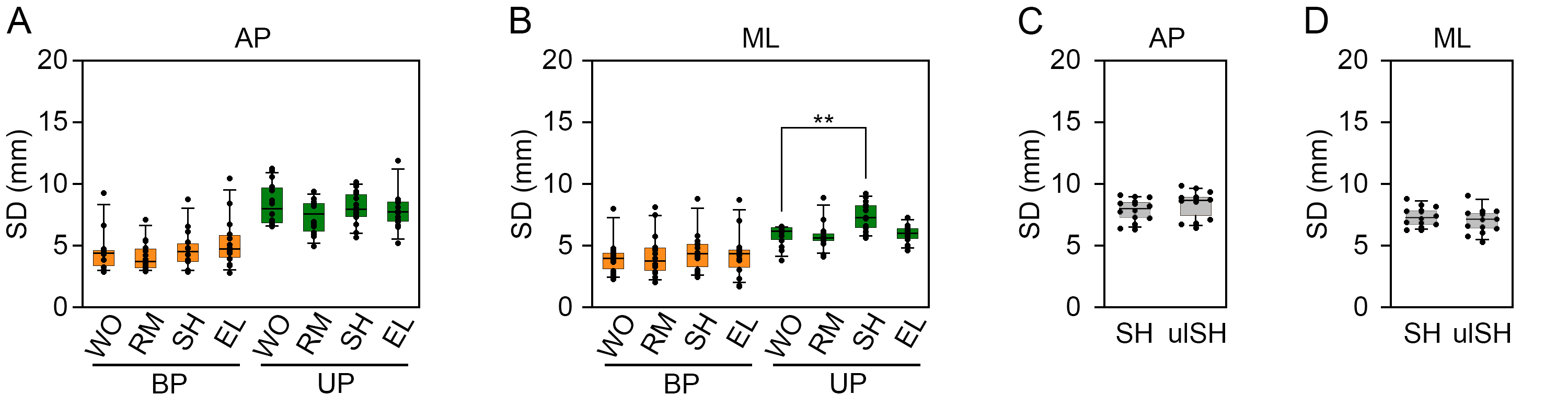

Supplement: S1 Fig — (A) The standard deviations of COP displacement in the anteroposterior (AP) direction under the presentation of the WO-, RM-, SH-, and EL-type visual targets during BP (orange) and UP (green) standing. (B) The standard deviations of COP movement in the mediolateral (ML) direction under the presentation of the WO-, RM-, SH-, and EL-type visual targets during BP (orange) and UP (green) standing. (C, D) The standard deviations of COP movement in the anteroposterior (AP, C) and mediolateral (ML, D) directions under the presentation of the SH- and ulSH-type visual targets. The box plots represent the median, first and third quartiles (boxes), and fifth and 95th percentiles (whiskers). The number of participants: n = 14. Statistical differences were analyzed using Friedman’s analysis of variance followed by multiple Wilcoxon’s signed-rank test with Bonferroni correction. Abbreviations: AP, anteroposterior; BP, bipedal; ML, mediolateral; SD, standard deviation; UP, unipedal. Statistical significance is indicated by asterisks: * P < 0.00833, ** P < 0.00167. (TIF) [file pone.0276119.s002.tif]
