# Supplementary material for: Deterioration of postural control due to the increase of similarity between center of pressure and smooth-pursuit eye movements during standing on one leg
Source: PLoS One. 2022 Oct 13;17(10):e0276119. doi: 10.1371/journal.pone.0276119 (PMC9560487; doi:10.1371/journal.pone.0276119)
Supplement: Attachment — Submitted filename: Response to comments 220928R.docx [file pone.0276119.s004.docx]

**Reply to comments**

**Journal Requirements:**

Please review your reference list to ensure that it is complete and correct. If you have cited papers that have been retracted, please include the rationale for doing so in the manuscript text, or remove these references and replace them with relevant current references. Any changes to the reference list should be mentioned in the rebuttal letter that accompanies your revised manuscript. If you need to cite a retracted article, indicate the article’s retracted status in the References list and also include a citation and full reference for the retraction notice.

**Reply:** According to the requirement, we checked the reference list. No retracted papers were cited.

**Additional Editor Comments:**

The reviewers are very porsitive about the revision, and only a few minor comments remain to be addressed (see below). I hope you can fix these points soon.

**Reply:** We appreciated for the positive evaluation. According to reviewer’s comments, we have totally amended our manuscript. We hope that the revised manuscript is now acceptable for publication in *PLoS One*.

**Response to Reviewers’ Comments**

**Reviewer #1:** All queries have been addressed adequately and correctly by the authors. The additions in the text as well as the corrections in the figure are satisfactory.

**Reply:** We appreciated for the positive evaluation.

**Reviewer #2:**

**Comment 1:** - Page 6 line 120-121: How are you calculating the “amplitude of the COP displacement”? Instead of stating it multiple times in the results, (lines 260 to 261 and 374-376), consider stating this in the methods.

**Reply:** The authors apologize for the missing description. According to comment, we have added the methodology of calculating the amplitude of the COP displacement in the Methods section as follows:

(page 8, line 181-184) The relative proportion of the area under the spectral plots of power in each frequency bandwidth was calculated. The sum of the area under the spectral plots of amplitude in each frequency bandwidth was designated as the amplitude of the COP displacement.

**Comment 2:** - Page 6 line 122-123: Consider adding the word “were” after “There are two reasons why participants”.

**Reply:** The authors apologize for the missing description. According to comment, we have corrected the sentence as follows:

(page 6, line 122-123) There are two reasons why participants were tested 1 week apart.

**Comment 3:** - Page 17: Consider removing the word “one” after XX-type throughout the discussion.

**Reply:** The authors appreciate the helpful comment. According to comment, we have totally deleted the word “one” after XX-type throughout the discussion section.

(page 17, line 396 & 399; page 22, line 511 & 514) WO-type

(page 17, line 403) SH-type

**Comment 4:** - Page 18 line 406: Consider adding the word “been” after “It has also”.

**Reply:** According to comment, we have corrected the sentence as follows:

(page 18, line 408-410) It has also been reported that the effect of saccade on the root mean square in the ML direction is smaller than that of smooth pursuit, and the significant improvement in upright stability is not observed due to the difference of experimental conditions

Comment 5: - Page 19 line 446: Consider adding the word “the” as such “In the present study”.

**Reply:** According to comment, we have corrected the sentence as follows:

(page 19, line 448-449) In the present study, we revealed that the amplitude of COP displacement was larger during UP standing than during BP standing.
